# Unsupervised Learning with Generative Adversarial Network for Automatic Tire Defect Detection from X-ray Images

**DOI:** 10.3390/s21206773

**Published:** 2021-10-12

**Authors:** Yilin Wang, Yulong Zhang, Li Zheng, Liedong Yin, Jinshui Chen, Jiangang Lu

**Affiliations:** 1 State Key Laboratory of Industrial Control Technology, College of Control Science and Engineering, Zhejiang University, Hangzhou 310027, China; zjuwyl@zju.edu.cn (Y.W.); 12032053@zju.edu.cn (Y.Z.); zhengli2020@zju.edu.cn (L.Z.); 21832038@zju.edu.cn (L.Y.); lujg@zju.edu.cn (J.L.); 2 Zhongce Rubber Group Co., Ltd., Hangzhou 310018, China; 3 Zhejiang Laboratory, Hangzhou 311121, China

**Keywords:** generative adversarial network, tire defect detection, anomaly detection, image reconstruction, memory-augmented module

## Abstract

Automatic defect detection of tire has become an essential issue in the tire industry. However, it is challenging to inspect the inner structure of tire by surface detection. Therefore, an X-ray image sensor is used for tire defect inspection. At present, detection of defective tires is inefficient because tire factories commonly conduct detection by manually checking X-ray images. With the development of deep learning, supervised learning has been introduced to replace human resources. However, in actual industrial scenes, defective samples are rare in comparison to defect-free samples. The quantity of defective samples is insufficient for supervised models to extract features and identify nonconforming products from qualified ones. To address these problems, we propose an unsupervised approach, using no labeled defect samples for training. Moreover, we introduce an augmented reconstruction method and a self-supervised training strategy. The approach is based on the idea of reconstruction. In the training phase, only defect-free samples are used for training the model and updating memory items in the memory module, so the reproduced images in the test phase are bound to resemble defect-free images. The reconstruction residual is utilized to detect defects. The introduction of self-supervised training strategy further strengthens the reconstruction residual to improve detection performance. The proposed method is experimentally proved to be effective. The Area Under Curve (AUC) on a tire X-ray dataset reaches 0.873, so the proposed method is promising for application.

## 1. Introduction

**Figure 1:**
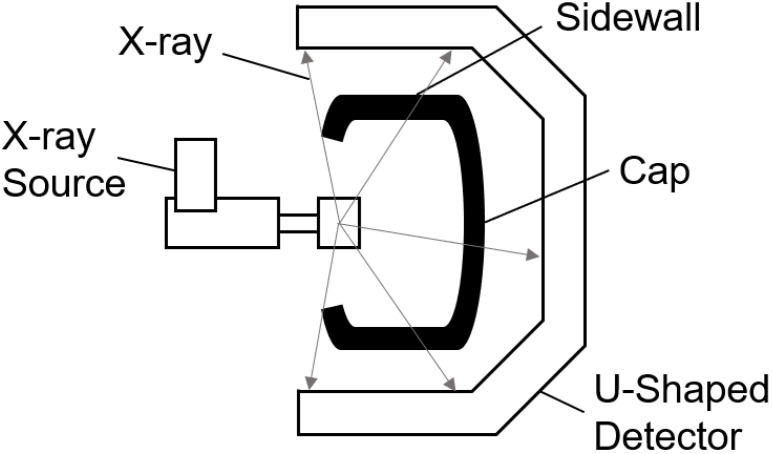
How the imaging equipment works.

**Figure 2:**
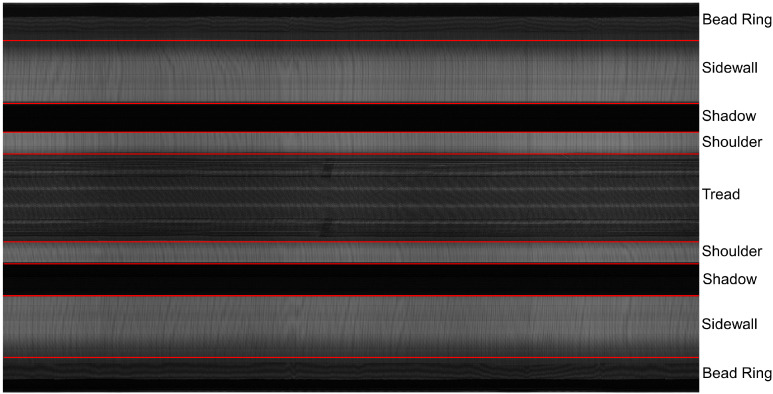
A horizontally displayed entire defect-free X-ray image, divided into five areas: bead ring, sidewall, shadow, shoulder and tread.

**Figure 3:**
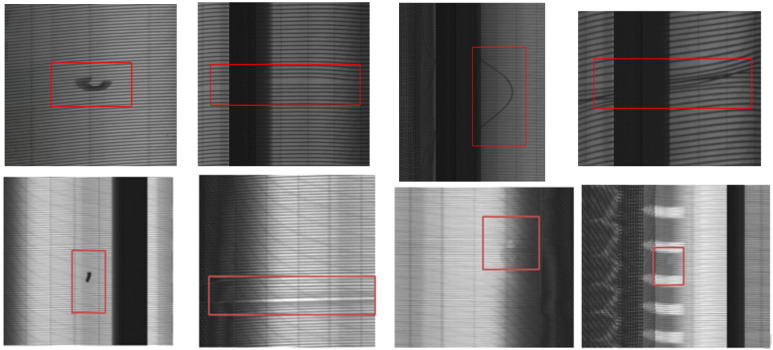
Typical defects in tire X-ray images.

Defect detection of industrial products is an important research content in the field of machine vision. It is a technology that uses machine vision equipment to obtain images and judge whether there are defects in images. Due to the complex production environment in the tire manufacturing process, there may exist various defects, such as impurities, blisters, and other issues. These quality issues are difficult to detect through surface detection. Thus, an X-ray sensor is commonly used to inspect the inner structure of tire. In tire industry, it is a crucial process for detecting defective tires through X-ray images [1]. This procedure can effectively prevent tires with quality issues from entering the market and reduce the risk of road problems [2]. During X-ray imaging, a tire is first conveyed to a lead shield room. Then, the door is closed. Next, the tire is hung on a tire expansion mechanism which is responsible for holding up and rotating the tire by $360^{\circ }$. Meanwhile, an X-ray source stretches into the tire and emits X-rays. A u-shaped detector receives the X-rays and performs imaging. Finally, the tire is delivered out from the lead shield room and the whole process ends. The imaging equipment sketch is shown in Figure 1. Figure 2 is an entire defect-free X-ray image displayed horizontally. An X-ray image can be divided into five areas: bead ring, sidewall, shadow, shoulder, and tread. The black areas in Figure 2 are shadow areas in imaging. They can be ignored during inspection. In the production process, various types of defects may occur. Typical defects in tire X-ray images are given in Figure 3.

At present, defect detection of tire factories mainly relies on experienced workers judging with by naked eye. However, there are many problems with manual defect detection, such as time complexity and strong subjectivity, which is unconducive to large-scale industrial production. To tackle these problems, in recent years, automated defect detection has become a hotspot in this field. Traditional defect detection methods for industrial products first preprocess images via histogram equalization, filtering, or image binarization to obtain separated information of the foreground and background; then, the defect detection is performed using statistical learning methods [3,4]. The traditional methods have a narrow scope of application and are ineffective for some defects with small scale and large background interference. In addition, they have poor robustness and high pertinence. When the scene changes, the designed parameters tend to require adjustment. With the development of image processing algorithms and computer vision, many new solutions have been proposed for industrial defect detection [5]. Deep learning (DL)-based defect detection methods can be divided into—(1) fully supervised learning (FSL) methods which rely on the classification of labeled samples; (2) semi-supervised and unsupervised learning methods which rely on training on defect-free samples. At present, FSL methods have made several achievements, some of which have even been incorporated into the actual production process to save labor and reduce costs. FSL methods include techniques based on representation and metric learning. In representation learning, the problem of defect detection is considered a classification task in the field of computer vision. Some methods from the field of computer vision are directly applied to industrial defect detection, such as the classification network [6,7], detection network [8,9,10] and segmentation network [11,12]. In metric learning, whether two input images belong to the same category is judged via the Siamese network [13,14]. However, the performance of FSL methods depends heavily on the amount of labeled data. Instead, methods based on defect-free samples only use a small amount of labeled data along with a large amount of unlabeled data to train and test the defect detection model. To date, there are few studies on defect detection methods based on defect-free sample learning applicable to industrial production.

In the tire industry, X-ray images acquired from X-ray machines can be utilized for automated inspection of tire defects. The inspection process usually involves two steps: image acquisition and defect detection. The image acquisition procedure is mainly responsible for the digital image capture of defect-free and defective samples, and generally, an X-ray machine can be used. The defect detection procedure is performed to give detection results directly. In this paper, we mainly concentrate on the defect detection step, which is considered challenging in tire quality inspection.

In actual industrial scenarios, the occurrence frequency of many defect types is extremely low and the number of samples is insufficient for training the existing models. In the production process, defects belonging to a classified category with unseen shapes or defects belonging to a new unclassified category may occur. The effect of FSL methods on the detection of such defects is poor. In addition, a large number of defect-free samples, which FSL cannot fully use, can be obtained in industrial production. Therefore, unsupervised methods based on defect-free samples are more suitable for industrial defect detection scenarios. Instead of extracting features of defect samples, we manage to memorize patterns of defect-free samples and then pick out a handful of outliers. This problem can be defined as an anomaly detection problem. We demonstrate a problem definition of anomaly detection: given a training dataset $D=\{x^{1},x^{2},\cdots ,x^{N}\}$, which contains plenty of normal samples, a model *f* is optimized to learn the distribution $p_{x}$ during training. Then in the test phase, given a new example *x*, an anomaly score A(x) is computed to identify if it is similar to the training data in D. The computation of anomaly scores depends on the choice of anomaly detection methods. The major concern of this study is how to more efficiently mine the information on a large number of defect-free samples and train the anomaly detection model to identify outliers.

In this work, we are motivated to address the aforementioned drawbacks of FSL methods. We propose a **G**enerative adversarial network (GAN)-based **A**nomaly **D**etection method in which a **M**emory-**A**ugmented module is implemented (MAGAD). In anomaly detection methods, no labeled defect samples are required to train models, whereas the defect-free samples can be fully utilized. Our motivation is to learn features of defect-free samples during training. For this purpose, two techniques are introduced as follows. First, a GAN-based reconstruction model is designed. A structure of autoencoder (AE) is employed as the generator. With a large number of defect-free images as training samples, two-dimensional (2D) pixel matrices will be transformed into embeddings as the latent representation, which is compressed information of the input images. Particularly, a memory-augmented module is added to the latent space of the autoencoder to store the learned embeddings as memory items. These memory items are taken out as the input of the decoder part to generate images as similar to the original input defect-free images as possible. Second, a simple but effective image-inpainting strategy is utilized to learn the compressed representation of defect-free images. The model is forced to generate complete images with corrupted input through the context. The encoder needs to understand the entire image and learn a representation which captures the semantics of visual structures [15]. By the above two efforts, defect-free images are fully utilized to learn the representation, and therefore they will be well reconstructed. In contrast, the reconstruction performance of the unseen anomalies during the test phase will be extremely low. By comparing the reconstruction performance, the anomaly detection task is achieved. The main contributions of this paper are as follows.

- We propose a novel unsupervised anomaly detection method which has the advantage of good compatibility for industrial defect detection. This method does not rely on large quantity of labeled defect images, saving labor and time simultaneously.
- We introduce a memory-augmented module and an image-inpainting strategy to learn the representation of defect-free images. Specifically, the memory-augmented module is an effective component for feature learning and memorization, whereas the image-inpainting strategy assists the model with context understanding. They both strengthen the reconstruction residuals for anomaly detection.
- We show that the proposed method leads to significantly finer reconstructed images. It is superior to classic anomaly detection algorithms on industrial defect detection tasks. In addition, ablation studies are conducted to verify the effectiveness of introduced modules.

The remainder of this paper is organized as follows. In Section 2, we review the related works. In Section 3, we introduce the overall structure of the model and analyze the principle and function of each module in detail. In Section 4, we present the experiment results of a tire X-ray dataset for defect detection; we further compare them with other existing methods and conduct ablation studies. Finally, Section 5 presents a summary of this research and some future research directions.

## 2. Related Work

### 2.1. Semi-Supervised and Unsupervised Defect Detection Method

Owing to the particularity of industrial scenes, semi-supervised and unsupervised methods with less demand for labeled defect samples are more suitable, but the research on these methods is still insufficient. Di et al. [16] built a semi-supervised generative adversarial network based on the trained convolutional autoencoder called CAE-SGAN, achieving good results in the steel surface defect detection. Gao et al. [17] proposed a semi-supervised learning method to classify steel surface defects using a convolutional neural network to predict unlabeled samples and gain pseudo labels. Images with pseudo labels are taken as training samples for further training. In 2017, Schlegl et al. [18] proposed a deep convolutional generative adversarial network—AnoGAN, performing the image inspection in latent space. The abnormal score was computed via the difference in both image and latent space. Then in 2019, Schlegl et al. [19] proposed f-AnoGAN based on AnoGAN. F-AnoGAN introduced an encoder module and obtained a better effect than AnoGAN. In 2018, Ganomaly [20], proposed by Akçay et al., and the improved method Skip-Ganomaly [21] can generate clear reconstructed images and recognize them by analyzing the latent vector in latent space. Mei et al. [22] proposed a multiscale AE reconstruction method based on the Gaussian pyramid proposed, achieving a good performance on the detection of blocky defects. Zhao et al. [23] combined the AE with GAN. By designing artificial defects as the input of the network, the model they proposed was trained to eliminate defects and reconstruct defect-free images. Then, the original and reconstructed images were input to the local binary pattern algorithm [24] for computing the difference. However, the aforementioned methods still have drawbacks. Some of them have relatively simple structure design and are only suitable for datasets of small-size images. The others do not have a satisfactory reconstruction effect, so extra classifiers need to be introduced and trained with the labeled data to detect defects. In addition, the DL training methods based on defect-free samples have certain robustness when training large-scale data and in some cases erroneously retain defects that should not appear in the test phase, thereby resulting in misjudgment. In our work, we propose a model to rectify these shortcomings.

### 2.2. Attention Mechanism and Memory-Augmented Module

The structure of a memory-augmented module is based on an attention mechanism. The attention mechanism imitates the process of biological visual recognition. By matching external information (input features) with internal experience (memory bank), the attention mechanism realizes the extraction of refined information for a region of interest. In 2014, with the development of DL, the Google Mind team [25] combined an attention mechanism with a recurrent neural network model and made significant progress in image classification tasks. In 2017, Vaswani et al. [26] proposed Transformer, which discards CNN and RNN totally. It consists of self-attention modules and feed forward neural networks. Based on Transformer, several applications have been put forward in the field of image classification [27], object detection [28], and time-series forecasting [29]. Memory-augmented network is designed based on the attention mechanism and has attracted increasing interest. Santoro et al. [30] proposed a meta learning method with a memory-augmented neural network to solve the problem of few-shot learning. Li et al. [31] investigate the application of memory units on deep generative models, using external memory and attention mechanism to capture local detailed information. Gong et al. [32] added memory network into autoencoder, effectively inhibiting the reconstruction performance of anomalies. In our work, we design a memory network as a module in the generative adversarial network for reconstruction and defect detection.

## 3. The Proposed Method

In industry, there are a large number of defect-free samples, whereas some types of defect samples are insufficient. It is difficult to achieve ideal results when using the technique of training a binary classification network to detect defects. Considering this problem, we adopt the idea of anomaly detection. First, defect-free samples are trained to extract features, and then these features are decompressed to obtain reconstructed images. By comparing the original and reconstructed images, we can determine whether the original image is defective, owing to the fact that the network only memorizes the features of the defect-free samples. When defect images pass through the generator, defective features will be discarded, thereby generating defect-free images, so the original input and reconstructed images will be different. With residual computation methods, the differences can be quantified as abnormal scores and anomalies can be identified.

**Figure 4:**
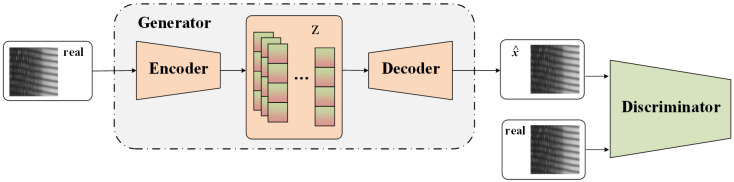
Structure of basic reconstruction GAN.

**Figure 5:**
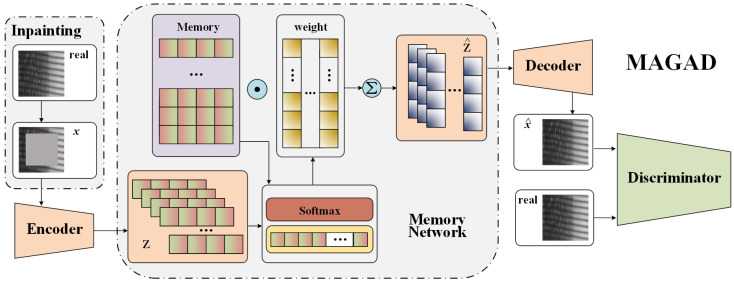
Structure of MAGAD.

The architecture of the defect detection is described in detail in this section. The overall structure of basic reconstruction GAN and MAGAD are shown in Figure 4 and Figure 5. In the training phase, the network takes defect-free images as input to obtain the reconstruction ability. In the test phase, the trained generator is used as the reconstruction model, taking the test dataset—containing images of both defect and defect-free samples—as input. Then, the similarity between the original and reconstructed images is calculated to detect defects and obtain detection results.

### 3.1. Model Training

As a widely used model in semi-supervised learning and unsupervised learning, AE can be used to complete the task of image reconstruction. It has good performance in image denoising, anomaly detection, and data dimensionality reduction. However, in the process of encoding and decoding, some information will be lost. AE cannot always discard information properly. As a result, reconstructed images may retain some defects or become too fuzzy. Since Goodfellow et al. [33] proposed GAN in 2014, it has developed rapidly and has gradually displaced AE. GAN can generate clearer images than AE. In this section, we design a novel image reconstruction model based on GAN, as is shown in Figure 5.

#### 3.1.1. GAN

The basic model of GAN consists of two parts: generator G and discriminator D. Traditional GAN often performs the task of image generation from a random signal. However, we need GAN to remove the defects of the image and restore a defect-free image. The structure of basic reconstruction GAN is shown in Figure 4. In the training phase, the input of the model is a defect-free image *x*, and then the image is encoded as a corresponding feature code *z*. Next, the feature code is decoded as a reconstructed image $\hat{x}$ with the same size as the original input *x*. Discriminator D is essentially a classifier. Its input is the original image *x* and reconstructed image $\hat{x}$. The structure of discriminator D is the same as the encoder part of the generator. The classification result is obtained based on the downsampled feature map to determine whether the input image is the original (real) or reconstructed (fake) image. The results can drive the generator backward to generate more realistic reconstruction images. In this article, we design the encoder and decoder based on the generator and discriminator of deep convolutional GAN (DCGAN) [34]. The input size used in this article is 256×256 and the dimension of the encoding vector is 1024. The structure specifications are listed in Table 1 and Table 2.

#### 3.1.2. Memory-Augmented Module

**Figure 6:**
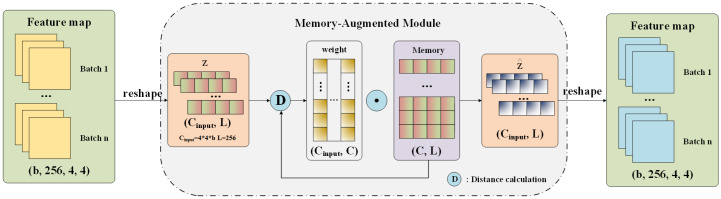
Details of the memory-augmented module.

In theory, the trained model only memorizes the pattern of defect-free samples. However, in the test phase, a part of defects will still be restored, resulting in smaller reconstruction residuals. Therefore, to increase the reconstruction residual of the defect images, we introduce a memory-augmented module to enhance the memorization of the pattern of defect-free samples. The memory-augmented module consists of memory items to store the prototypical encoded patterns of defect-free samples and an attention-based addressing operator unit for accessing the memory. During training, the memory contents are updated to record the prototypical features. Given a testing sample, the model performs reconstruction merely using these recorded patterns. In other words, features about anomaly patterns will be dropped. As a result, the reconstructed images tend to be close to defect-free samples, resulting in large residuals on anomalies. Figure 6 presents details of the memory-augmented module.

The memory-augmented module used in this work is a matrix *M* with dimension C×L, where *C* is the storage capacity of the module and *L* is the length of the input feature vector. The memory-augmented module is located between the encoder and decoder, storing the typical features of defect-free images of tires during training.

To insert the memory-augmented module into the designed AE, the last convolutional layer of the encoder (Table 1) is discarded and the feature map with dimension 256×4×4 is obtained. The feature map is a 4D tensor (b,256,4,4), where *b* represents batch size. It is then reshaped into a 2D tensor $\left(C_{input},L\right)$, as the input of the memory-augmented module. In the training phase, the memory-augmented module receives the feature code $Z\in R^{C_{input}*L}$ from the output of the encoder, where *L* is the length of the vector, corresponding to the length of the vector in memory *M*. The distance between *Z* and the past image features stored in the memory-augmented module can be calculated by

(1) $$D_{z∼m}=\frac{Z\cdot M^{T}}{∥M∥∥Z∥}.$$

Then, the distance is normalized by row, that is, for each input feature vector, the weight $W_{i}$ between the feature vector *i* and all memory items is calculated according to the distance by

(2) $$W_{i}=\frac{\exp\left(D_{ij}\right)}{\sum_{j}\exp\left(D_{ij}\right)}.$$

In some cases, the complex combination of memory items may constitute an anomaly pattern. To prevent this situation, the threshold ε, which takes the value of 0.0025, is introduced to ensure the sparsity of the weight [32] each time the weight is calculated and a weight less than the threshold is set to zero, that is

(3) $$W_{i}^{\prime }=\left\{\begin{array}{cc} W_{i}, & \;\mathrm{if}\;W_{i}>\varepsilon .\\ 0, & \;\mathrm{otherwise}.\; \end{array}\right.$$

Equation (3) can be rewritten as follows:

(4) $$W_{i}^{\prime }=\frac{f\left(W_{i}-\varepsilon \right)\cdot W_{i}}{abs\left(W_{i}-\varepsilon \right)+e},$$

where $f\left(x\right)$ is a ReLU function and *e* is a small constant $10^{-12}$ to prevent the denominator from being zero. Then, the weight $W^{\prime }$ is used to reconstruct the feature-coding vector $\hat{Z}$:

(5) $$\hat{Z}=W^{\prime }\cdot M.$$

Similarly, the first layer of the decoder (Table 2) is discarded and $\hat{Z}$ obtained by the memory-augmented module is directly input into the decoder to reconstruct images after being reshaped into (b,256,4,4). Memory in the memory module (Figure 6) is initialized as a C×L tensor. The memory items are set as trainable parameters during the training phase, so they will be updated along with other layers. Intuitively, representations stored as memory items in the memory are updated during training.

In the test phase, feature vectors *z* of defect images will be the input of the memory module. It is hypothesized that feature vectors $z_{1}$ and $z_{2}$ are containing the defect and background information, respectively. After going through the memory-augmented module, the background information can be retained better since the information contained in $z_{2}$ has been stored in the module. Meanwhile, the information in $z_{1}$ has not been recorded; it has a large gap from the background information, resulting in the weights *W* being mostly set to zero. After going through the module, the information about the defects is discarded and the vector is reconstituted into a feature vector similar to the background information. More exactly, after decoding, defect areas on the reconstructed images will no longer retain.

#### 3.1.3. Image Inpainting

To better obtain features from datasets of a large number of defect-free images, we employ the concept of an image-inpainting task. In the image-inpainting task, there is a blocky occlusion on an image, so predictions need to be made for missing parts through the context. Only if the model really understands the image and learns the features of the image can the missing area be effectively inpainted. This is also the goal of self-supervised learning—mining as many features as possible with limited data. In 2016, Pathak et al. [15] used the method of image inpainting to learn the context information in an image, which also achieved the purpose of feature learning while successfully inpainting the image.

**Figure 7:**
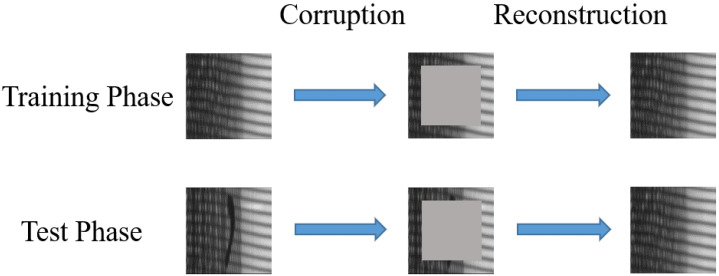
Procedure of image inpainting.

In this study, the technique of image inpainting is applied in defect detection (Figure 7). In the training phase, the pixels of the center area of an input defect-free image are replaced with random Gaussian noise, resulting in some features being corrupted. The network is trained to predict missing parts with the remaining pixels, thereby making the network have a better grasp on the overall structure of defect-free images. The same operation is performed in the test phase, corrupting the features of the defect, which is more conducive to reconstructing defect-free images and further augmenting the differences between the original and reconstructed images.

#### 3.1.4. Loss Function

To improve the reconstruction performance and the accuracy rate of detection, the memory-augmented module and image-inpainting training method are incorporated into the basic GAN. As such, the training process is more complicated. In this section, the loss function designed for model training is introduced.

Since the model adopts a structure based on GAN [33], the loss function of the model can be divided into two parts—the loss of the generator and discriminator.

The loss of the generator includes image reconstruction, sparsity, and basic generative adversarial losses. The reconstruction loss consists of two parts—residual and structural similarity losses—to impel the generator to have a good learning of contextual features of input data. The two parts of loss are exerted because they focus on different image properties. The residual loss focuses on pixel-level similarity and the structural similarity loss focuses on patch-level similarity. The residual loss is obtained by calculating the mean absolute error of the original and reconstructed image as follows:

(6) $$Loss_{res}=\frac{1}{mn}\sum_{r=0}^{m-1}\sum_{c=0}^{n-1}\left|x\left(r,c\right)-G\left(x\right)\left(r,c\right)\right|,$$

where *m* and *n* are the height and width of the original image, respectively; x(r,c) and G(x)(r,c) are the values of a certain pixel of the original and reconstructed image, respectively. We adopt L1 loss since L1 yields less blurry results than L2 [35].

Typical methods of calculating image similarity include mean squared error (MSE), peak signal-to-noise ratio (PSNR) [36], structural similarity (SSIM) [37], information fidelity criterion (IFC) [38], visual information fidelity (VIF) [39], and some texture-based methods. MSE and PSNR only consider the difference between pixel values and are poor to evaluate the difference and similarity in human visual effects [40]. IFC, VIF, and texture-based methods have satisfactory performance but require high computational complexity. SSIM has better performance of similarity evaluation in human visual effects than MSE and PSNR with relatively lower computational complexity than IFC, VIF, and texture-based methods. It is also proved in subsequent experiments that the reconstruction performance is satisfactory by using SSIM loss introduced in this paper. The evaluation function of SSIM is as follows:

(7) $$\mathrm{SSIM}\left(x,y\right)=\frac{\left(2\mu_{x}\mu_{y}+c_{1}\right)\left(2\sigma_{xy}+c_{2}\right)}{\left(\mu_{x}^{2}+\mu_{y}^{2}+c_{1}\right)\left(\sigma_{x}^{2}+\sigma_{y}^{2}+c_{2}\right)},$$

where $\mu_{x}$ and $\mu_{y}$ are the mean values of *x* and *y*, respectively; $\sigma_{x}$ and $\sigma_{y}$ are the variances of *x* and *y*, respectively; $\sigma_{xy}$ is the covariance of *x* and *y*; $c_{1}={\left(k_{1}L\right)}^{2}$ and $c_{2}={\left(k_{2}L\right)}^{2}$; *L* is the range of pixel values using 255 for 8-bit images; $k_{1}$ and $k_{2}$ are generally set to 0.01 and 0.03, respectively; $c_{1}$, $c_{2}$, and $c_{3}$ are constants used to prevent destabilization of the system when the denominator approaches zero. An 11×11 sliding window with a stride of 1 is used to extract image patches. Then, the SSIM value of the corresponding region is calculated. Assuming that the total number of sliding windows on the entire image is *N*, the SSIM evaluation function for the entire image is given by:

(8) $$\mathrm{SSIM}\left(x,y\right)=\frac{1}{N}\sum_{j=1}^{N}\mathrm{SSIM}(x_{j},y_{j}).$$

The SSIM loss function is designed as follows:

(9) $$Loss_{str}=1-\mathrm{SSIM}\left(x,y\right).$$

To prevent overfitting during the training phase and prevent identity mapping while reconstructing the defect image, an additional sparsity loss is introduced for the weight matrix in the memory-augmented module, given by

(10) $$Loss_{spr}=E_{w∼p}\left[-\log\left(W\right)\right],$$

where *W* is the weight matrix in the memory-augmented module.

Generative adversarial loss is used to compute the distance of features in the discriminator and thus optimize the generation of the generator to produce a reconstruction image that is more similar to the original image and able to cheat the discriminator. From some current findings [41], the training instability of GAN can be reduced by feature matching [42]. By extracting the feature maps of original and reconstructed images output in the discriminator, we incorporate the distance of the two feature maps into loss to make them as identical as possible. Therefore, we define the generative adversarial loss as follows:

(11) $$Loss_{adv}=E_{x∼p}[|f\left(x\right)-f\left(G\left(x\right)\right)|],$$

where *x* and G(x) represent the original and reconstructed images, respectively; f(x) represent the downsampling operation of the discriminator.

Eventually, the loss function of the generator is defined as follows:

(12) $$\begin{array}{c} Loss_{G}=\lambda_{1}Loss_{res}+\lambda_{2}{Loss}_{str}+\lambda_{3}{Loss}_{spr}+\lambda_{4}{Loss}_{adv}, \end{array}$$

where $\lambda_{1}$, $\lambda_{2}$, $\lambda_{3}$, and $\lambda_{4}$ are all weighting factors.

The discriminator is used to discriminate whether the input image is an original or a reconstructed image produced by the generator. We use a least-squares loss to measure, given by:

(13) $$Loss_{D}=\frac{1}{2}E_{x∼p}\left[{\left(D\left(x\right)\right)}^{2}\right]+\frac{1}{2}E_{\hat{x}∼\hat{p}}\left[{\left(D\left(G\left(x\right)\right)-1\right)}^{2}\right].$$

### 3.2. Model Test

The defect detection in this article is based on the idea that the reconstruction residual of a defect image is much larger than that of a defect-free image. To measure the similarity of the original and reconstructed images, thereby obtaining the discriminative basis for recognizing defects, it is necessary to calculate the image reconstruction residual. Commonly used image reconstruction loss functions including L2 loss [43], mean squared error [44], SSIM [45], etc., belong to the methods that directly compare the images before and after reconstruction. It is also possible to perform defect detection based on differences between the distributions of features of defect-free and defect samples using methods such as GAN models proposed by Schlegl et al.—AnoGAN [18] and f-AnoGAN [19], support vector machine classifiers proposed by Liu et al. [46] for defect classification, and Ganomaly [20] proposed by Akçay et al.

In this article, we decide to employ the former scheme—calculating the similarity of images before and after reconstruction. Next, we introduce the calculation method of the anomaly scores designed in this study. The calculation of anomalies in the test set is slightly modified based on the basic SSIM introduced earlier for defect detection objectives. Typically, the calculation of basic SSIM is computing the mean value of sliding windows of images in the size of 11×11. However, in the defect detection task, averaging across defects is easy to leave small defects out if their sizes are small compared to the entire image, so the operation of taking the average of the sliding window area is changed to taking the maximum value, i.e.,

(14) $$\mathrm{SSIM}\left(x,y\right)=\max_{N}\;\mathrm{SSIM}\left(x_{j},y_{j}\right).$$

For each image in the test dataset, we calculate an anomaly value $s_{i}$ for each sample. Then, we normalize values across all test set to be in the interval [0,1]. Extreme values in the test phase appeared in the experiment, so the use of linear normalization will lead to an extremely uneven distribution of abnormal scores. Therefore, the sigmoid normalization method is employed. We obtain the result as the final abnormal score using the formula

(15) $$s_{i}^{\prime }=\frac{1}{1+e^{-a(s_{i}-b)}},$$

where *a* is an adjustable parameter that controls the slope of the sigmoid curve and *b* is the mean value of samples in the test dataset; $s_{i}^{\prime }$ is the final anomaly score, which is used to identify if the current underdetermined sample is a defective one.

## 4. Experiment Results

In this section, we conduct experiments on the proposed model and compare it with other traditional anomaly detection methods. We also carry out ablation studies about the impact of different components.

### 4.1. Datasets and Evaluation Metric

**Figure 8:**
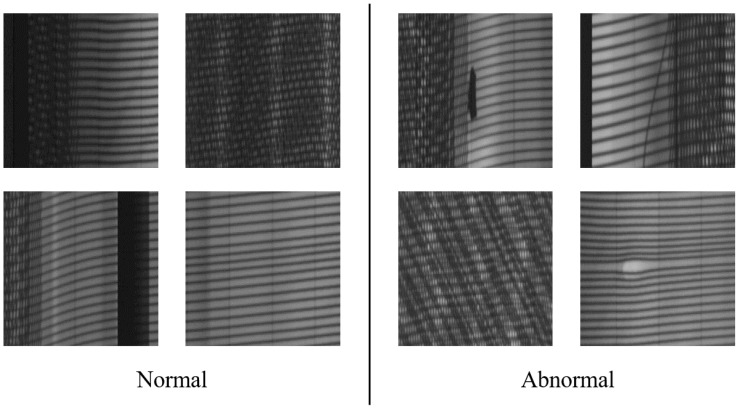
Normal and abnormal examples of the dataset used in this paper. They are grayscale images in the shape of 256×256×1.

The experimental dataset employed in this study was obtained from tire X-ray images of an industrial production line. Tire X-ray images were taken by the X-ray machine in the factory and annotated by experienced workers. After cropping the primitive high-resolution images (Figure 2), the dataset was obtained. The size of images is 256×256×1. The dataset includes 21 types of examples in total such as foreign matters, blisters, and so on, covering all of the types provided from the tire factory. They are integrated into two types—normal and abnormal types—for our research. The two types of images are shown in Figure 8. To simulate the condition that there are a large number of defect-free samples and a small number of defect samples in the real industrial scenario, 20,000 defect-free images were selected as the training set to train the reconstruction model, whereas 2077 defect images and 3947 defect-free pictures were selected as the test set for testing the defect detection effect.

We use accuracy, recall, and AUC (Area Under Curve) as the measurement for performance evaluation. Accuracy is calculated as the proportion of the number of samples that are classified correctly over the total number of samples. Recall is calculated as the proportion of the number of positive samples that are classified correctly over the total number of positive samples. AUC is obtained by calculating the area under the Receiver Operation Characteristic (ROC) with a varying threshold, whereas ROC is plotted according to the True Positive Rate and False Positive Rate.

### 4.2. Implementation Details

We adopt the encoder and decoder module of DCGAN [34] as the backbone network of GAN; the structure is shown in Table 1 and Table 2. We train the model with an Adam optimizer on the training dataset for 200 epochs. We set the initial learning rate to 0.0002 and momentums $\beta_{1}=0.5$, $\beta_{2}=0.999$ with batch size 128. We set the weight factors of $Loss_{G}$ as $\lambda_{1}=50$, $\lambda_{2}=5$, $\lambda_{3}=0.005$, and $\lambda_{4}=3$. Owing to the fact that the number of samples for training is sufficient, we did not use data augmentation during training. We implement our model using Pytorch 1.7.0 and Python 3.6. Our experiments are run on a workstation with Nvidia RTX 3090 GPU and Intel Xeon-E5 2.50GHz CPU, RTX 3090 GPU containing 328 tensor cores.

### 4.3. Results

#### 4.3.1. Detetion Results

We compare the proposed model with several conventional and deep learning-based methods for general anomaly detection, including basic autoencoder, f-AnoGAN [19], DSEBM [47], and Ganomaly [20]. Among these, We adopt the structure of Table 1 and Table 2 for basic autoencoder method to detect 256×256 images. We obtain the detection results of each method (Table 3) using accuracy, recall, and AUC as the evaluation indexes. Clearly, from the quantitative results, the proposed method outperforms the other methods.

**Figure 9:**
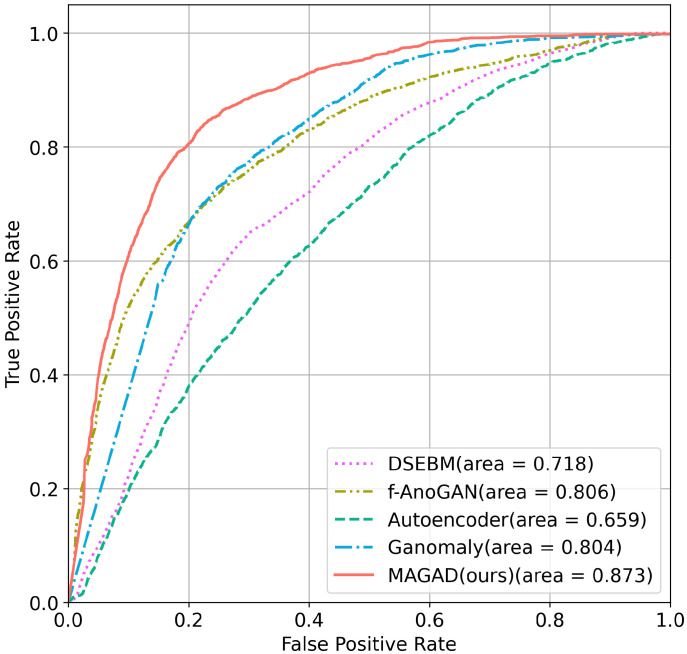
Comparison of ROC curves among five models.

ROC curves of the five methods on the tire X-ray test dataset are plot for comparison. As shown in Figure 9, the AUC of the proposed method reaches 0.873. The introduced memory-augmented module and image-inpainting strategy are significantly more effective for detection.

In addition, extensive comparisons among the variants, i.e., the memory size and the size of corrupted blocks in inpainting strategy, are conducted to verify the impact of the parameters chosen in the proposed method.

**Figure 10:**
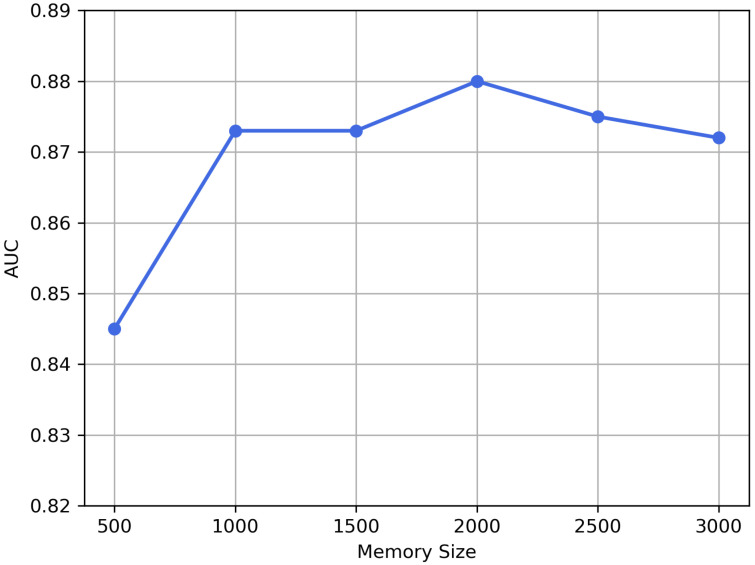
Impact of the size of memory on AUC.

**Memory size.** The memory is an important part in the memory-augmented module. We study the robustness of the proposed method to the memory size, that is, the storage capacity *C* of the memory module. We conduct experiments by using different memory size settings and show the AUC values in Figure 10. From Figure 10, memory in the size of 500 cannot effectively memorize the representations. Sizes of above 1000 enable the model to produce ideal reconstruction results. A larger size will contribute to higher computational complexity with approximate performance, so 1000 is enough.

**Figure 11:**
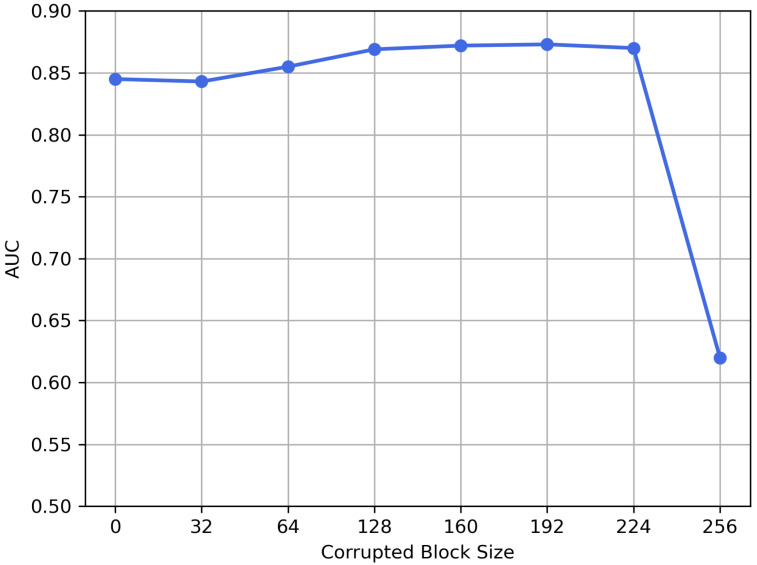
Impact of the size of corrupted blocks on AUC.

**Size of corrupted blocks.** We investigate the performance of the inpainting strategy under different sizes of corrupted blocks, as shown in Figure 11. In fact, size 0 means doing reconstruction without inpainting strategy. The larger size means a larger corrupted area in an image and more information requires being inferred through the context by the reconstruction model. From Figure 11, with the expansion of the area, AUC is gradually increasing. Notably, size of 256 means that the input of the reconstruction model is entirely noise, so there will not be an ideal reconstruction result, showing a poor performance (AUC = 0.62). The final size is set to 192, according to the experiments.

#### 4.3.2. Reconstruction Results of Defect Images

**Figure 12:**
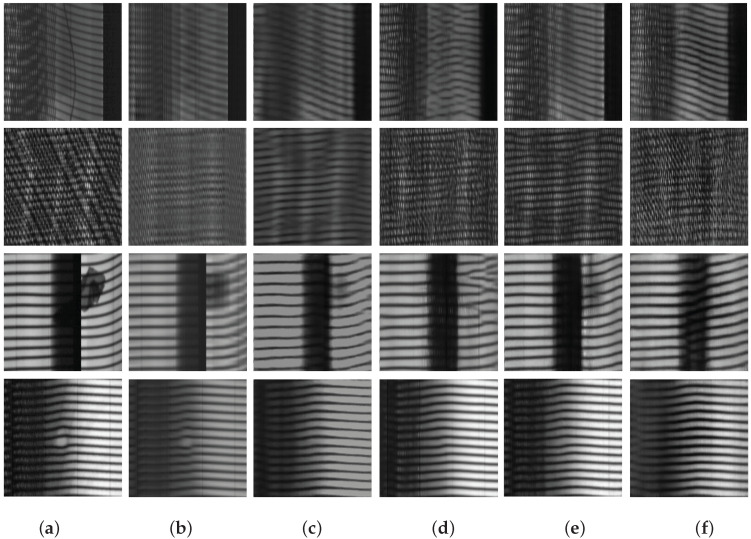
Images from top to bottom are defects of 0-Degree-Scatter, Belt-Joint-Open, Foreign Matter, and Blister, respectively. Images from left to right, are the (**a**) original images and reconstruction results of the model (**b**) PCA, (**c**) AE, (**d**) GAN, (**e**) GAN-Mem, and (**f**) MAGAD.

In this subsection, to further prove and explain why MAGAD is effective, we turn to verify the reconstruction performance. We compare the proposed model with four conventional reconstruction methods including a traditional method—principal component analysis (PCA), AE, basic GAN, GAN model with a memory module, and the proposed MAGAD. The details are as follows. (1) Model A: PCA reconstruction method. PCA is a data dimension reduction method in multivariate statistical analysis. It is used for image reconstruction when considering the input image as a 2D matrix to extract main features. The extracted features are used for reconstruction; (2) Model B: AE reconstruction method. The AE can achieve dimension reduction similar to PCA. Its difference from PCA is that it is a nonlinear dimension reduction method. The structures employed in the experiments of AE are the same as the structure shown in Table 1 and Table 2; (3) Model C: basic GAN reconstruction method. It takes the structure of the AE in model B as the generator part and an encoder as the discriminator; (4) Model D: GAN reconstruction method incorporating a memory-augmented module based on model C without an image-inpainting strategy; (5) Model E: the proposed method—MAGAD. We select four typical tire defects, including 0-Degree-Scatter, Belt-Joint-Open, Foreign-Matter, and Blister, to demonstrate the reconstruction results (Figure 12).

- **0-Degree-Scatter**

As shown in the first line of Figure 12, the five models remove the scatter successfully. However, PCA and AE both blur the tread pattern, whereas GAN without a memory module distorts the cords. The introduction of the memory module and image-inpainting strategy tackles these problems. Sharpness is maintained and defect areas are removed simultaneously.

- **Belt-Joint-Open**

As shown in the second line of Figure 12, for these disordered patterns, it is challenging for PCA to extract features because PCA compresses each image individually. PCA is unable to find regularities in these images. Similar to defects of 0-Degree-Scatter, AE fails to reserve the tread pattern and even makes the reconstruction image fuzzy. When the structure of generator and discriminator is adopted, the reconstruction performance is significantly improved.

- **Foreign-Matter** & **Blister**

When it comes to Foreign-Matter, the most common defects in tire defect detection, all but MAGAD, are unsatisfactory, as shown in the third line of Figure 12. Notably, from Figure 12e,f, the addition of image inpainting strategy helps corrupt features of defects. Pixels in the center area are inferred through the context, so defects are removed totally. Blister in the last line of Figure 12 is similar to the Foreign-Matter.

To sum up, methods based on data-driven training perform better than traditional statistical learning methods like PCA. The existence of discriminators in GAN solves the problem of fuzzy reconstruction. The introduction of memory module avoids image distortion. The image inpainting strategy helps remove defect areas thoroughly. These introduced techniques contribute to reinforcing the reconstruction residuals of defects, thereby improving the detection accuracy rate. The reconstruction ability of the model is of great significance, so according to the aforementioned analysis, it is as expected that our model shows a satisfactory detection result as presented in the previous subsection.

### 4.4. Ablation Study

We conduct ablation studies to investigate the role of each component in our MAGAD model in Table 4. We experiment to study the importance of each component by removing the other one. As shown in Table 4, removing either the memory module or the image inpainting strategy will degenerate the performance, which demonstrates the effectiveness of our proposed method MAGAD. Based on the model basic GAN, we first add an image inpainting strategy to obtain model GAN-Inpainting, which is an improvement over basic GAN. Further incorporating the memory module results in our MAGAD performing best in all variants. This indicates the importance of the memory module. We also create a model GAN-Mem by excluding the image inpainting strategy from MAGAD and the performance drops a lot, showing that the self-supervised learning strategy plays a crucial role in our proposed model. This result conforms to the aforementioned analysis in method introduction and the reconstruction results we previously showed. The influence of the two modules on the result is very close. A combination of the two brings about a leap in performance.

### 4.5. Runtime Test

We present a comparison of runtime during the test phase, using methods mentioned in Section 4.3.1. As shown in Table 5, except for f-AnoGAN [19] with much longer running time, the other four methods have quite similar runtime. Although MAGAD takes a little more time than Autoencoder and Ganomaly [20] owing to its model complexity, we believe this is acceptable because of its significant performance improvement.

## 5. Conclusions

In summary, this article presents a novel unsupervised defect detection algorithm—MAGAD—for tire X-ray images based on reconstruction residuals under an industrial scene. The algorithm does not rely on training a network with a large number of annotated defect samples; it only requires the use of defect-free samples for training the network. The addition of a memory-augmented module allows features of defect-free images to be held in the items of the memory bank and reduces the probability that defects are reconstructed incorrectly. The introduction of an image-inpainting strategy helps the network understand the structure of an image in depth during training. Moreover, it reduces the defective information input of defect images at the test phase, thereby further reducing the probability of reconstructing defects. Experimental data show that the addition of the memory-augmented module and image-inpainting strategy significantly enhances the reconstruction effect and improves the performance.

In actual industrial scenes, images are usually high-resolution ones. For high-resolution image inputs, the training of GAN is still difficult. How to complete the reconstruction and defect detection for images with high resolution will be a future research direction. Further, existing anomaly detection algorithms for defect inspection can only accomplish binary classification detection tasks, so determining how to improve the algorithms and solve multi-classification problems is also a future research direction.

## Figures and Tables

**Table 1:** The structure of encoder.

Type	Filters	Size/Stride	Output
Convolutional	8	4×4/2	8×128×128
LeakyReLU			
Convolutional	16	4×4/2	16×64×64
BatchNorm + LeakyReLU			
Convolutional	32	4×4/2	32×32×32
BatchNorm + LeakyReLU			
Convolutional	64	4×4/2	64×16×16
BatchNorm + LeakyReLU			
Convolutional	128	4×4/2	128×8×8
BatchNorm + LeakyReLU			
Convolutional	256	4×4/2	256×4×4
BatchNorm + LeakyReLU			
Convolutional	1024	4×4/1	1024×1×1

**Table 2:** The structure of decoder.

Type	Filters	Size/Stride	Output
Transposed Convolutional	256	4×4/1	256×4×4
BatchNorm + Relu			
Transposed Convolutional	128	4×4/2	128×8×8
BatchNorm + Relu			
Transposed Convolutional	64	4×4/2	64×16×16
BatchNorm + Relu			
Transposed Convolutional	32	4×4/2	32×32×32
BatchNorm + Relu			
Transposed Convolutional	16	4×4/2	16×64×64
BatchNorm + Relu			
Transposed Convolutional	8	4×4/2	8×128×128
BatchNorm + Relu			
Convolutional	3	4×4/2	3×256×256
Tanh			

**Table 3:** Detection results of five models.

	Accuracy(%)	Recall(%)	AUC
Autoencoder	62.3	58.9	0.659
f-AnoGAN [19]	75.4	67.0	0.806
DSEBM [47]	63.9	72.6	0.718
Ganomaly [20]	74.9	70.1	0.804
**MAGAD (ours)**	**79.8**	**83.1**	**0.873**

**Table 4:** Ablation studies on different components of our method.

Method	Memory Module	Image Inpainting	AUC
Basic GAN	×	×	0.804
GAN-Inpainting	×	✔	0.848
GAN-Mem	✔	×	0.830
**MAGAD**	✔	✔	0.873

**Table 5:** Runtime test of five models.

Method	Mean Runtime Per Image (ms)
Autoencoder	10.08
f-AnoGAN [19]	19.14
DSEBM [47]	12.16
Ganomaly [20]	10.64
**MAGAD**	11.73

## Data Availability

Not applicable.
